# Root Canal Configuration of Burmese (Myanmar) Maxillary First Molar: A Micro-Computed Tomography Study

**DOI:** 10.1155/2021/3433343

**Published:** 2021-11-30

**Authors:** M. M. Kyaw Moe, H. J. Jo, J. H. Ha, S. K. Kim

**Affiliations:** ^1^Department of Conservative Dentistry, University of Dental Medicine, Mandalay, Myanmar; ^2^Department of Conservative Dentistry, Kyungpook National University, Daegu, Republic of Korea

## Abstract

**Aim:**

To investigate the root canal anatomy of Burmese (Myanmar) permanent maxillary first molar (BMFM) with micro-computed tomography. *Methodology*. One hundred and one extracted BMFMs were scanned by a SkyScan 1272 scanner (Bruker microCT, Belgium) and reconstructed with NRecon software (Bruker microCT). CTAn software (Bruker microCT) was used to create 3D models of root and internal canal anatomy, while CTVol software (Bruker microCT) was used to visualize 3D models. In each root, Vertucci's canal types, incidence and location of the lateral canal, incidence, location, and type of isthmus, and number and position of foramina were examined.

**Results:**

In 101 specimens, 83 (82.18%) mesiobuccal roots had multiple canals. The most common canal type is type IV (45.5%), followed by type II (17.8%) and I (17.8%) canals. Type III, V, VI, VII, and VIII canals are less than 10% in total. Seven additional canal types were seen for 10% in total. Fourteen (13.86%) distobuccal roots had multiple canals, and the predominant canal type is type I (86.1%), followed by type II (5.9%) and V (4%) canals. Three additional canal types were observed for 4% in total. All palatal roots possessed the simplest type I canal. Apical ramification occurred in 69 mesiobuccal roots (68.3%), 36 distobuccal roots (35.6%), and 37 palatal roots (36.6%). A total of 240 lateral canals were observed in 101 specimens. Each specimen had 2.38 ± 2.22 lateral canals on average. The highest incidence, 136 (56.67%) lateral canals, occurred in the mesiobuccal root, followed by 57 (23.75%) and 47 (19.58%) lateral canals from the distobuccal root and the palatal root, respectively. Each specimen had 6.17 ± 2.42 foramina. Mesiobuccal root had the highest incidence of apical foramina compared to other roots. Seventy-two mesiobuccal roots (71.29%) had isthmus, while only 7 distobuccal roots (6.93%) had isthmus somewhere along the root.

**Conclusions:**

The root canal anatomy of BMFM was quite complex, especially in the mesiobuccal root. The predominant canal type was Vertucci type IV in the mesiobuccal root and type I in the distobuccal and palatal roots. In addition, this micro-computed tomography study disclosed complemented canal types and a higher prevalence of lateral canal than the previous studies.

## 1. Introduction 

A thorough understanding of root canal anatomy (RCA) and its variation is extremely important for endodontic treatment [1]. Maxillary first molar (MFM) is one of the most frequently endodontically treated teeth [2, 3] and the posterior teeth with the highest endodontic failure rate [4]. The majority of MFMs have three roots and four canals [5]. Because of its internal anatomic intricacy, it has been extensively studied by various methods [5]. Historically, the clearing method was considered as the gold standard for three-dimensional (3D) study of human RCA; however, this method is not suitable to reveal the complex and fine anatomic features of RCA in the mesiobuccal root of MFM, and micro-computed tomography (*µ*CT) is currently becoming the reference method for this purpose [6]. Several studies used *µ*CT to investigate the RCA of MFM in different population [7], especially in mesiobuccal root, and showed more complex internal RCA than previously expected and higher incidence of the additional canal [6, 8–12] than previous studies that used traditional methods such as clearing, sectioning, or grinding [5]. Ethnicity, age, and gender differences might influence the diversity of RCA [12, 13]. One previous study also applied *µ*CT to study the RCA of MFM in the Egyptian population [7]. In 2001, Ng et al. studied the RCA of Burmese MFM by clearing method [14]. Burmese MFM possesses the complex RCA and shows additional Vertucci's canal types [14]. To date, there was no *µ*CT-based study for the RCA of Burmese MFM. Therefore, this study aimed to investigate the RCA of Burmese MFM by using the current gold standard *µ*CT analysis.

## 2. Materials and Methods

The ethics committee of the University of Dental Medicine, Mandalay, Myanmar, approved this study (Ethical/UDMM/2018/17). One hundred and one extracted human permanent MFMs with three distinct roots were selected from the Burmese population. Specimens with immature apex, apical root fracture, and previous endodontic treatment were not included in this study. Debris and stain on tooth surface were cleansed by hand and ultrasonic scalar. Specimens were stored in saline solution during the study period. Then, specimens were scanned by a SkyScan 1272 scanner (Bruker microCT, Kontich, Belgium) with 10 *µ*m voxel size, 125 *µ*A, 80 mV, 1 mm aluminum filter, 0.4° rotation steps, and 180° rotation. After reconstruction with NRecon software v1.6.1 (Bruker microCT), CTAn software v1.14.4 (Bruker microCT) was used to create 3D models of root and internal canal anatomy, while CTVol software v2.2.3 (Bruker microCT) was used to visualize these 3D models in color-coded and transparent models. The segmentation or thresholding of the scan dataset was performed by CTAn Software. The proper threshold value for enamel, dentine, and pulp in the crown and root was determined in the density histogram. Original grayscale images and segmented images were compared and checked to obtain accurate result. The global thresholding method was used. Thresholding, morphological operation, despeckle, bitwise operation, and 3D model tasks from the custom processing function tab in CTAn software were applied as necessary to produce the 3D models of the root and root canal systems. Then, these 3D models were visualized in color-coded and transparent modes by CTVol software. In each root, Vertucci's canal type, incidence and location of the lateral canal, incidence, location, and type of isthmus, and number and position of foramina were investigated. The main canal is defined as any canal extending from pulp chamber floor to apex and possessing its own or shared orifice and main apical foramen. The lateral canal is defined as any branch emerging from the main canal to communicate with the periodontium. Apical delta or ramification is defined as the presence of any branch, apart from the main canal, communicating the periodontium in the apical third of the root. Isthmus is defined as any communication, sheet- or tube-like structure, between main canals whether completely or incompletely connecting to each other. The isthmuses observed were three-dimensionally categorized into six types, such as sheet complete type: a narrow sheet-like structure connecting the main canals anywhere, sheet incomplete type: a narrow sheet-like structure extending from either of main canals but not completely connecting each other at any point, tubular complete type: a tube-like structure extending from either of main canals connecting each other at any point, tubular incomplete type: a tube-like structure extending from either of main canals but not completely connecting each other at any point, mixed complete type: a narrow sheet- and tube-like structure together connecting the main canals anywhere, and mixed incomplete type: a narrow sheet- and tube-like structure extending together from either of main canals but not completely connecting each other at any point [15].

## 3. Results

### 3.1. Root Canal Types and Incidence of Apical Ramification

Vertucci's canal types with additional canal types observed in mesiobuccal, distobuccal, and palatal roots are summarized in Table 1.

**Figure 1:**
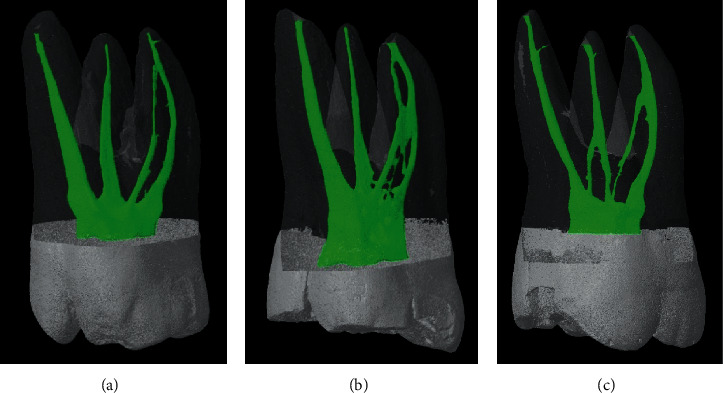
Burmese maxillary first molars with Vertucci's type II canal in the mesiobuccal root. (a) Mesiobuccal root shows Vertucci's type II canal with the lateral canal at the apical third, while distobuccal and palatal roots show Vertucci's type I without lateral canal. (b) Mesiobuccal root shows Vertucci's type II canal with sheet complete isthmus at the coronal and middle thirds, while palatal and distobuccal roots show Vertucci's type I canal. (c) Both mesiobuccal and distobuccal roots show Vertucci's type II canal, while palatal root shows Vertucci's type I canal with a lateral canal at the apical third.

**Figure 2:**
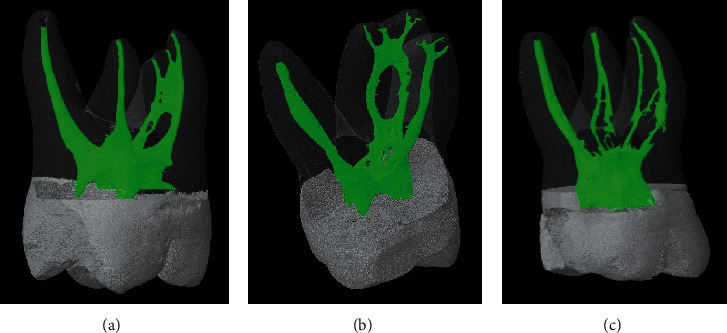
Burmese maxillary first molars with Vertucci's type IV canal in the mesiobuccal root. (a) Mesiobuccal root shows Vertucci's type IV canal, while distobuccal and palatal roots show Vertucci's type I canal. A sheet complete isthmus with a lateral canal was seen at the middle and apical third of the mesiobuccal root. (b) Mesiobuccal root shows Vertucci's type IV canal with sheet complete isthmuses at the apical and coronal third. A lateral canal was originating from the isthmus in the apical third of the mesiobuccal root. Distobuccal and palatal roots show Vertucci's type I canal. (c) Mesiobuccal root shows Vertucci's type IV canal with a mixed complete isthmus at the middle third, and distobuccal root discloses Vertucci's type II canal with a tube complete isthmus at the middle third. Palatal root shows Vertucci's type I canal.

**Figure 3:**
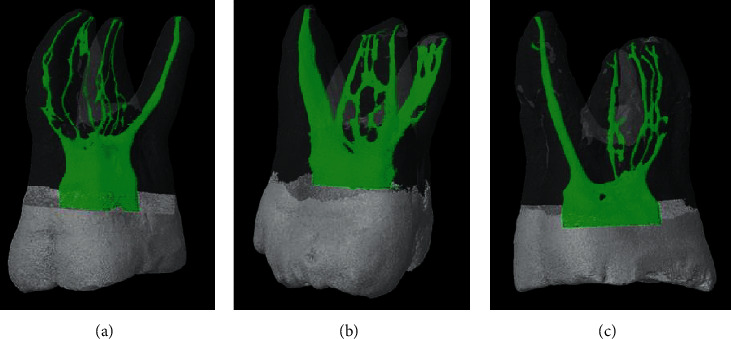
Burmese maxillary first molars with supplemental root canal configurations. (a) Mesiobuccal root shows Vertucci's type VIII canal, while distobuccal and palatal roots reveal an additional canal type (3-2) and Vertucci's type I canal, respectively. Sheet complete isthmus is connecting two additional mesiobuccal canals at the apical region of the mesiobuccal root, and tube complete isthmus is communicating between canals in the distobuccal root at all thirds. (b) Mesiobuccal root shows an additional canal type (4-4), while the distobuccal and palatal roots reveal an additional canal type (1–3) and Vertucci's type I canal, respectively. Sheet complete type isthmus is connecting four mesiobuccal canals at the coronal, middle, and apical regions of mesiobuccal root. (c) Mesiobuccal root shows Vertucci's type VIII canal with lateral canals at the apical third and sheet complete isthmus at the middle third. Both distobuccal and palatal roots reveal Vertucci's type I canal with a lateral canal at the apical third.

In the mesiobuccal root, 83 (82.18%) specimens had multiple canals. The most common canal type is type IV (45.5%) canal, followed by type II (17.8%) and I (17.8%) canals (Figures 1–3). Type III, V, VI, VII, and VIII canals are less than 10% in total. Seven supplemental canal types, (3-2), (4-4), (2-3), (3-1), (3-2-4), (1-2-1-2), and (1-2-4-1-2), were also seen in mesiobuccal root for 10% in total.

In the distobuccal root, 14 (13.86%) specimens had multiple canals and the predominant canal type is type I canal (86.1%), followed by type II (5.9%) and V (4%) canals. Three supplemental canal types, (1-3), (3-2), and (2-1-2-1-2), were also observed for 4% in total. Other types were not observed.

In the palatal root, 100% of the specimen possessed the simplest type I canal.

Apical ramification occurred in 69 mesiobuccal roots (68.3%), 36 distobuccal roots (35.6%), and 37 palatal roots (36.6%).

### 3.2. Incidence of Lateral Canal and Location of Lateral Canal

A total of 240 lateral canals were observed in 101 MFMs. Each specimen had 2.38 ± 2.22 lateral canals (0.87 ± 0.98, 0.39 ± 0.76, 0.55 ± 0.94, 0.47 ± 0.70, and 0.17 ± 0.53 lateral canals from mesiobuccal, additional mesiobuccal, distobuccal and palatal canals, and isthmus, respectively). The highest incidence, 136 (56.67%) lateral canals, occurred in the mesiobuccal root, followed by 57 (23.75%) and 47 (19.58%) lateral canals from the distobuccal root and the palatal root, respectively (Table 2). Nineteen (18.81%) specimens had no lateral canal, while 17 (16.83%) specimens had lateral canals at all roots. The number of specimens with lateral canals in roots and with different number of the lateral canal is expressed in Tables 3 and 4.

### 3.3. Number and Position of Canal Foramina

Each specimen had 6.17 ± 2.42 foramina (1.92 ± 0.98, 0.98 ± 1.01, 1.63 ± 0.97, 1.47 ± 0.70, and 0.17 ± 0.55 foramina from mesiobuccal, additional mesiobuccal, distobuccal, and palatal canals, and isthmus, respectively). Incidence of foramina in roots, root canals, and isthmus, and specimen with different number of foramina is expressed in Tables 5 and 6. Mesiobuccal root had the highest incidence (49.60%) of foramina compared to two other roots.

### 3.4. Incidence, Location, and Type of 3D Isthmus

The distribution of 3D isthmus types in the mesiobuccal and distobuccal roots at different root thirds is expressed in Table 7. Mesiobuccal root revealed a higher number of isthmuses compared to distobuccal root in all root thirds. Seventy-two mesiobuccal roots (71.29%) had isthmus, while only 7 distobuccal roots (6.93%) had isthmus somewhere along the root.

## 4. Discussion

This study investigated the root canal configurations of Burmese MFM by *µ*CT analysis. In the endodontic literature, Weine's and Vertucci's classifications are commonly and classically used in RCA studies [12]. Previous studies [10, 12] showed that Vertucci's classification had wider coverage to classify the complex RCA of human teeth than Weine's classification. Recently, a 4-digit system and another new system were introduced to classify the RCA of human teeth [7, 16]. In the 4-digit system, the first three digits represent the canal number at the coronal limit of the respective thirds only and the last digit behind the slash is for the number of main apical foramina. We tried to use these systems; however, these systems did not fit our study and may not reflect the actual complexities of human RCA. In our opinion, Vertucci's classification with modification for complementary canal types is suitable for our study and applied in this study.

It was reported that the prevalence of mesiobuccal root with multiple canals in laboratory studies by traditional methods (60.5%) is higher than that of clinical studies (54.7%) [5]. Six previous *µ*CT studies showed a high prevalence of mesiobuccal root with multiple canals (71.3–100%) [6, 8, 10, 12, 17]. Only two *µ*CT studies showed a relatively lower prevalence of mesiobuccal root with multiple canals (46.6% and 55.6%) [7, 11]. The prevalence of mesiobuccal root with multiple canals in this study (82.18%) is within the range of that (46.6–100%) from previous *µ*CT studies and is quite similar to that of Korean (71.3%), American (100%), and Italian (100%) but different from Egyptian (46.6%) and Japanese (55.6%) [6, 12, 17]. However, one recent cone-beam computed tomography (CBCT) clinical study in Egyptian at 133 *µ*m voxel size stated that 74.55% of the mesiobuccal root of MFM had additional canals [18]. It might be due to the sample size, the study method, the different tribes, or different canal classification systems used, for example, the 4-digit system in Egyptian study and modified Weine's canal classification in the Japanese study. Additionally, 82.18% of mesiobuccal root in this study had multiple canals, which is higher than that of the previous studies by clearing method (70%) for Burmese MFM [14] and (67.2%) for Thai MFM [19].

Success in endodontic treatment depends on the adequate cleaning, shaping, and obturation of the entire root canal system. The frequent failure of endodontic treatment was likely due to the failure to treat the additional canals [20]. A previous CBCT clinical study showed that teeth with a missed canal were 4.38 times more likely to be associated with apical lesions and that the incidence of missed canals was highest in the MFM [21].

The ability of the CBCT method to detect the MB2 canal mainly depends on its voxel size. The results may be varied from study to study due to the different spatial and contrast resolutions used. The currently available voxel size for the best resolution of CBCT analysis is 76 *µ*m. In one previous clinical study, even with the 76 *µ*m voxel size, three MB2 canals were not seen on the CBCT images but were identified after troughing under magnification and illumination [22]. CBCT is a very useful state-of-the-art 3D imaging technique for clinical cases; however, it still has the limitation to detect the fine anatomic structure of complex root canal systems, like MB2 canal.

Although the clearing method was previously considered as the best for the root canal anatomy study, it has some weaknesses, like the distortion of specimen during decalcification and subsequent difficulty in dye penetration, resulting in the inadequate presentation of fine anatomic structures including additional canals [6]. Nowadays, *µ*CT analysis can disclose more real 3D complex anatomic structures and the actual prevalence of additional canals in the RCA of MFM, especially in mesiobuccal root, compared to traditional methods, grinding, sectioning, or clearing techniques [6]. Because of radiation hazards, *µ*CT analysis can only be applied in in vitro studies. From these results, the prevalence of additional canals in the mesiobuccal root of MFM in different populations by traditional laboratory methods should be revised by *µ*CT analysis.

The majority (70%) of the inhabitants of Myanmar possess Mongoloid traits [23]. In this study, the prevalence of type IV canal (45.5%) is highest, followed by type I canal (17.8%) and type II canal (17.8%) in the mesiobuccal root, showing the typical Mongoloid trait [12], whereas Caucasian population shows more type II canal [24]. Also, the previous studies stated that type IV is predominant over type II canal in the mesiobuccal root of MFM of Burmese [14], Indian [24], Japanese [11, 25], Korean [26], and Thai [19], which conforms to our result. The higher prevalence of type IV (2 orifices with 2 foramina) compared to type II (2 orifices with single foramen) is the endodontic significance for the presence of multiple portals of exits and more microleakages readily [27]. Considering these facts, the clinician should take attention to find the additional canal in the mesiobuccal root of Burmese MFMs during the treatment until it is excluded.

In mesiobuccal roots, the present *µ*CT study exhibited more additional canal types, (3-2, 4-4, 2-3, 3–1, 3-2-4, 1-2-1-2, and 1-2-4-1-2), for 10%, than the previous clearing study, (2-1-2-1, 2-3, and 3-2), for 3.3% [14]. This might be attributed to the better ability of *µ*CT, nondestructive and higher resolution 10 *µ*m, to reproduce the fine and complex anatomic structures accurately by compensating the drawbacks of the clearing methods, distortion of specimen, and limited dye penetration into minute structures.

Variation in additional canal occurrence in the distolingual root was less frequently seen in this study. However, the prevalence of distobuccal root with multiple canals in this *µ*CT study is 13.9%, which is higher than that of previous clearing studies (5.5% and 1.9%) [14, 19]. Only one additional canal type (2-3) for 1.1% occurred in the previous clearing study [14], while the present *µ*CT study identified three additional canal types, (1–3), (3-2), and (2-1-2-1-2), for 1%, 2%, and 1%, respectively. So, the relatively more complex RCA of distobuccal roots was disclosed by the benefit of *µ*CT compared to the previous clearing study that showed a more simple type I canal [14].

All palatal roots (100%) in this study possessed the simplest type I canal configuration and lack of complexity and isthmus, which is similar to the previous findings [14, 19], showing less variation of this root. In contrast, one previous *µ*CT study demonstrated that 1.2% of palatal roots of Egyptian had more than one canal and it might be due to the different canal classification systems used and different tribes [7].

In this study, the highest incidence of apical ramification was observed in the mesiobuccal root (68.3%) possessing 56.7% of lateral canals and 49.6% of main and lateral canal foramina compared to the distobuccal root (35.6%) with apical ramification possessing 23.75% of lateral canals and 26.64% of main and lateral canal foramina and the palatal root (36.6%) with apical ramification possessing 19.58% of lateral canals and 23.76% of main and lateral canal foramina. These complex anatomic structures were commonly located at the apical region and similar to previous findings [10, 14, 28]. In contrast, the present *µ*CT study identified 240 lateral canals in 101 MFMs, mainly in the apical portion of the mesiobuccal root, which is much higher than only 22 lateral canals in 90 MFMs by the clearing method [14]. This discrepancy could be explained by the better performance of *µ*CT technology. These minute structures harboring microbes and/or necrotic tissues could not be shaped, cleaned, and properly filled and may cause postoperative pain and subsequent failure [20]. By these facts, clinicians should pay attention to clean these fine and complex anatomic structures by using the copious amount of proper chemical disinfectant with agitation in the very apical portion of mesiobuccal root during cleaning and shaping.

In this study, isthmuses were more common in the mesiobuccal root compared to the distobuccal root, especially at the coronal and middle thirds of roots, and this is agreed with the previous results [6, 14]. In 101 specimens, the mesiobuccal root had 149 isthmuses whereas the distobuccal root had only 10 isthmuses, which is in contrast to the previous study [7]. Regarding the occurrence of isthmus, 71.29% of mesiobuccal roots and 6.93% of distobuccal roots had isthmus somewhere along the root, which is much higher than that of the previous study by clearing technique for the same population [14]. The previous *µ*CT study [7] mentioned about the connecting canals for only 2.9% of specimens, which might be similar to the tube complete isthmus type in our study.

The isthmus observed in this study were three-dimensionally categorized into six types based on the previous classification [15]: sheet complete type, sheet incomplete type, tubular complete type, tubular incomplete type, mixed complete type, and mixed incomplete type. Two 3D isthmus classifications were introduced by two previous studies [29, 30]. Here, we also devised the new 3D isthmus types according to the nature of anatomic structure and connectivity, which is based on the previous one [15] and it might be more convenient and wide-ranging than the previous two classifications.

One hundred and one extracted Burmese MFMs, not specified by age and gender, were used for this *µ*CT analysis, and this might be the limitation of this study.

## 5. Conclusions

The results of this study reiterate that the root canal configuration of Burmese MFMs is quite complex, especially the mesiobuccal root possessing the highest incidence of additional canals, lateral canals and apical delta, and isthmuses among three roots. The predominant canal type was Vertucci type IV in the mesiobuccal root, and type I in the distobuccal and palatal roots. This *µ*CT study showed a more detailed and much complicated root canal feature of Burmese MFMs than the previous study with Indian ink and clearing technique. The previous RCA studies by clearing technique for other tribes need to be revisited by the current gold standard *µ*CT analysis.

## Figures and Tables

**Table 1:** Root canal types observed in Burmese maxillary first molar (*n* = 101)

		Mesiobuccal root	Distobuccal root	Palatal root
Vertucci's canal types		No. of specimens (%)	No. of specimens (%)	No. of specimens (%)
Type I (1)		18 (17.8)	87 (86.1)	101 (100)
Type II (2-1)		18 (17.8)	6 (5.9)	0 (0.0)
Type III (1-2-1)		1 (1.0)	0 (0.0)	0 (0.0)
Type IV (2-2)		46 (45.5)	0 (0.0)	0 (0.0)
Type V (1-2)		3 (3.0)	4 (4.0)	0 (0.0)
Type VI (2-1-2)		1 (1.0)	0 (0.0)	0 (0.0)
Type VII (1-2-1-2)		2 (2.0)	0 (0.0)	0 (0.0)
Type VIII (3)		2 (2.0)	0 (0.0)	0 (0.0)

Supplemental types	(1–3)	0 (0.0)	1 (1.0)	0 (0.0)
	(3–2)	4 (4.0)	2 (2.0)	0 (0.0)
	(4–4)	1 (1.0)	0 (0.0)	0 (0.0)
	(2–3)	1 (1.0)	0 (0.0)	0 (0.0)
	(3–1)	1 (1.0)	0 (0.0)	0 (0.0)
	(3-2-4)	1 (1.0)	0 (0.0)	0 (0.0)
	(1-2-1-2)	1 (1.0)	0 (0.0)	0 (0.0)
	(1-2-4-1-2)	1 (1.0)	0 (0.0)	0 (0.0)
	(2-1-2-1-2)	0 (0.0)	1 (1.0)	0 (0.0)

1-canalled root = type I; 2-canalled root = type II, III, IV, V, VI, and VII; supplemental types (1-2-1-2), (1-2-4-1-2), and (2-1-2-1-2), 3-canalled root = type VIII, supplemental types (1–3), (3-2), (2-3), (3–1), (3-2–4), and (4-4).

**Table 2:** Incidence of lateral canals from main root canals and isthmus

Root	Canals/isthmus	Root thirds	Each third	Each canal or isthmus	Each root
			Number of lateral canals (%)	Number of lateral canals (%)	Number of lateral canals (%)
Mesiobuccal root	Mesiobuccal canal	Coronal	0 (0)	88 (36.67)	136 (56.67)
		Middle	2 (0.83)		
		Apical	86 (35.83)		
	Mesiobuccal 2 and additional canal	Coronal	0 (0)	32 (13.33)	
		Middle	32 (13.33)		
		Apical	0 (0)		
	Isthmus	Coronal	0 (0)	16 (6.67)	
		Middle	2 (0.83)		
		Apical	14 (5.83)		

Distobuccal root	Distobuccal canal and additional canal	Coronal	0 (0)	56 (23.33)	57 (23.75)
		Middle	1 (0.42)		
		Apical	55 (22.92)		
	Isthmus	Coronal	0 (0)	1 (0.42)	
		Middle	0 (0)		
		Apical	1 (0.42)		

Palatal root	Palatal canal	Coronal	0 (0)	47 (19.58)	47 (19.58)
		Middle	0 (0)		
		Apical	47 (19.58)		

Total number of lateral canals (%)				240 (100)	

^*∗*^Sixteen lateral canals occurred from the isthmuses of the mesiobuccal roots, and only one lateral canal occurred from the isthmus of the distobuccal root.

**Table 3:** Number of specimens (*n* = 101) with lateral canals in roots

Roots with lateral canals	All roots	No lateral canal at all roots	Mesial root only	Distal root only	Palatal root only	Mesial and palatal roots	Mesial and distal roots	Distal and palatal roots
Number of specimens (%)	17 (16.83)	19 (18.81)	28 (27.72)	5 (4.95)	6 (5.94)	12 (11.88)	12 (11.88)	2 (1.98)

**Table 4:** Number of specimens (*n* = 101) with lateral canals

	Number of lateral canals									
	0	1	2	3	4	5	6	7	8	12
Number of specimens (%)	19 (18.81)	27 (26.73)	17 (16.83)	11 (10.89)	10 (9.90)	8 (7.92)	3 (2.97)	4 (3.96)	1 (0.99)	1 (0.99)

**Table 5:** Number and position of canal foramina

Root	Canals/isthmus	Root thirds	Number of foramina (%)	Total number of foramina (%)
Mesiobuccal root	Mesiobuccal canal	Coronal	0 (0)	309 (49.60)
		Middle	1 (0.16)	
		Apical	193 (30.98)	
	Mesiobuccal 2 and additional canals	Coronal	0 (0)	
		Middle	0 (0)	
		Apical	99 (15.89)	
	Isthmus	Coronal	0 (0)	
		Middle	2 (0.32)	
		Apical	14 (2.25)	

Distobuccal root	Distobuccal canal and additional canal	Coronal	0 (0)	166 (26.64)
		Middle	0 (0)	
		Apical	165 (26.48)	
	Isthmus	Coronal	0 (0)	
		Middle	0 (0)	
		Apical	1 (0.16)	

Palatal root	Palatal canal	Middle	1 (0.16)	148 (23.76)
		Apical	147 (23.60)	

Total number of foramina (%)			623 (100)	

^*∗*^Sixteen lateral canals occurred from the isthmuses of the mesiobuccal roots, and only one lateral canal occurred from the isthmus of the distobuccal root.

**Table 6:** Number of specimens (*n* = 101) with foramina

	Number of foramina in each specimen										
	3	4	5	6	7	8	9	10	11	13	16
Number of specimens (%)	6 (5.94)	24 (23.76)	18 (17.82)	18 (17.82)	11 (10.89)	6 (5.94)	7 (6.93)	5 (4.95)	4 (3.96)	1 (0.99)	1 (0.99)

**Table 7:** Incidence of 3D isthmus types at different levels of mesiobuccal and distobuccal roots

Isthmus types (3D	Mesiobuccal root			Distobuccal root		
	Coronal third (%)	Middle third (%)	Apical third (%)	Coronal third (%)	Middle third (%)	Apical third (%)
Mixed complete	18 (12.08)	10 (6.71)	7 (4.70)	1 (10)	0 (0.0)	1 (10)
Mixed incomplete	9 (6.04)	8 (5.37)	2 (1.34)	0 (0.0)	1 (10)	0 (0.0)
Sheet complete	11 (7.38)	21 (14.09)	14 (9.40)	0 (0.0)	0 (0.0)	2 (20)
Sheet incomplete	6 (4.03)	3 (2.01)	1 (0.67)	0 (0.0)	0 (0.)	0 (0.0)
Tube complete	3 (2.01)	7 (4.70)	4 (2.68)	2 (20)	1 (10)	0 (0.0)
Tube incomplete	9 (6.04)	6 (4.03)	10 (6.71)	0 (0.0)	1 (10)	1 (10)
Total isthmuses in each third	56 (37.58)	55 (36.91)	38 (25.50)	3 (30)	3 (30)	4 (40)
Total isthmuses in each root	149 (100)			10 (100)		

## Data Availability

The data used to support this study are provided within the article.
